# Age-related circadian rhythm and variability of large- and small-airway function in healthy non-smoking adults: Data from 7-day diurnal and nocturnal home monitoring using an electronic portable spirometer

**DOI:** 10.3389/fpubh.2022.946988

**Published:** 2022-10-17

**Authors:** Xue Zhang, Yingying Zhang, Yan Zhou, Dongning Yin, Chengjian Lv, Jinwang Lin, Wuping Bao, Min Zhang

**Affiliations:** Department of Respiratory and Critical Care Medicine, Shanghai General Hospital, Shanghai Jiao Tong University School of Medicine, Shanghai, China

**Keywords:** age, small airway function, variation, circadian rhythm, home monitoring

## Abstract

**Background:**

The aim of the study was to investigate the possible influencing factors of the large- and small-airway function variation in healthy non-smoking adults.

**Methods:**

Healthy non-medical non-smoking adults were enrolled in this prospective cohort study. Each participant took the portable spirometer test relying only on video teaching. Then conventional spirometry and bronchodilation test were conducted using a Jaeger spirometer, followed by 7-day diurnal and nocturnal home monitoring using a portable spirometer.

**Results:**

A drop in both large- and small-airway function began at about 25 years of age, and a rapidly decline at about 50 years. The CV of FEV_1_ (*r* = 0.47, *P* = 0.0082) and small-airway function variables correlated with age (*r* ≥ 0.37, *P* < 0.05 for both MEFs and MEFs/FVC), especially for evening small-airway function variables. The CV of large (4.666 ± 1.946, *P* = 0.002 for FEV_1_; 4.565 ± 2.478, *P* = 0.017 for FEV_3_) and small airways (10.38 ± 3.196, *P* = 0.031 for MEF50 and 11.21 ± 4.178, *P* = 0.023 for MMEF) was higher in the 45- to 60-year subgroup than in the 30- to 45-year and 18- to 30-year subgroups.

**Interpretation:**

Age was the main influencing factor of both central and peripheral airway function variability, especially for the small-airway function in the evening. The LLN of small-airway variables varies depending on the age and circadian rhythm. People older than 45 years should pay more attention to monitoring small-airway function in the evening, which will be helpful for early clinical detection of those at high risk for asthma.

**Trial registration number:**

ChiCTR2100050355.

## Highlights

- What factors influence the circadian rhythm and variability of both large- and small-airway function in non-smoking healthy adults?
- Pulmonary function varies with circadian rhythm day to day and seasonally, age, standing height, sex, and ethnicity; however, there is a relative dearth of information regarding the possible influencing factors of the pulmonary function variation over time in healthy adults, especially the circadian rhythm and variation of both large- and small-airway function.
- Age was the main influencing factor of both large- and small-airway function variability, especially for the small-airway function in the evening after 45 years, indicating people older than 45 years should pay more attention to monitoring small-airway function nocturnally, which will be helpful for early clinical detection of those at high-risk for asthma.

## Introduction

Asthma is a common chronic airway disease, affecting estimated 400 million people worldwide and 45.7 million people in China (1–3). Although asthma is prevalent, the misdiagnosis rate, especially underdiagnosis in mild asthma, is massive (3). Objective evidence of variable expiratory airflow limitation is a key component in the asthma diagnosis process (1). However, the heterogeneity of the asthma compound is achallenge in diagnosis. Moreover, lack of effective objective monitoring contributes to uncontrolled symptoms, acute exacerbation, and death due to asthma, which have a substantial impact on healthcare costs.

Asthma is also a highly rhythmic airway disease, with clinical symptoms, lung function, and airway hyperresponsiveness varies in a circadian rhythm, day to day, seasonally, as well as from year to year (4–7). Spirometry, including central and peripheral airway function, fulfills a pivotal role in diagnosis, treatment response, and acute attack monitoring of patients with asthma. While comparison of an individual spirometry result with an appropriate reference or predicted value may identify abnormal lung function, it is often more clinically valuable according to normal variation range of lung function over time in an individual's level (8).

A laboratory spirometer (Jaeger spirometer) is too large and inconvenient to carry and too expensive for timely monitoring of lung function. Although peak expiratory flow (PEF) monitoring has been strongly recommended to demonstrate diurnal variability in asthma (9), the peak flow meters (PFMs) also have some limits, including low adherence, low accuracy, insensitivity to changes, and lack of large- and small-airway function indicators (10, 11). Thus, a portable spirometer with good quality control and concordance similar to that of a Jaeger spirometer serves as essential equipment of normal variation range monitoring, and the all-round management of asthma, is needed. Several portable spirometers have been validated to date (12–18), but most of them only show large-airway variables including PEF, forced expiratory volume in 1 s (FEV_1_), and forced vital capacity (FVC). Small-airway function indicators, which are increasingly important in the diagnosis, treatment, and monitoring of patients with asthma, are lacking in those spirometers (19–23). In our previous prospective cohort study based on data from 7-day morning and evening home monitoring using an electronic portable spirometer (GOSPT2000), we had validated GOSPT2000 is a reliable device and can serve as an alternative to a Jaeger spirometer for dynamic monitoring of lung function.

Recently, researchers have emphasized the significance of time (the fourth dimension) in asthma diagnosis. We have also investigated healthy individuals' circadian rhythm and variation features of large- and small-airway function in a previous study and found the FEV_1_, FVC, and FEV_3_ in the morning were higher than those at night, while no significant day–night difference was observed in small-airway variables. Pulmonary function varies with age, standing height, sex, and ethnicity; however, there is a relative dearth of information regarding the possible influencing factors of the pulmonary function variation over time in healthy adults, especially the circadian rhythm and variation of both large- and small-airway function.

The aim of this study was to investigate the possible influencing factors of the large- and small-airway function variation in healthy adults; whether professional training could improve the performance of a portable spirometer and whether factors such as age, sex, height, body mass index (BMI), or education degree affected the portable spirometer outcomes after professional training were also evaluated.

## Materials and methods

### Participants

This is a prospective cohort study approved by the Ethics Committee of Shanghai General Hospital, Shanghai Jiao Tong University (No. 2021KY073). We recruited participants from healthy adult volunteers, and 36 participants who met the inclusion criteria, had preserved lung function, and homogeneous with respect to gender, age, and educational levels—were enrolled. Informed consent was obtained for all the subjects.

To be included in the study, participants had to meet the inclusion criteria given as follows: 18–65 years old, no clinical symptoms within 8 weeks, normal comprehensive medical examination report (including routine laboratory tests such as complete blood counts, biochemistry tests, tumor markers such as carcinoma embryonic antigen and alpha fetal protein, B-ultrasonic examination, electrocardiography, and chest high-resolution computed tomography scan) within 1 month, no smoking history in their lifetimes, no allergic medical history such as allergic rhinitis or allergic dermatitis, no chronic respiratory disease, no previous pulmonary function test experience, and no medical background.

Subjects were excluded if they had systemic diseases, including the presence of unstable cardiovascular status; diabetes; gastroesophageal reflux; nausea; vomiting; abdominal pain; stress urinary incontinence; surgery of the chest, abdomen, or eye within the past 2 weeks; history of syncope associated with forced exhalation; unsuitable for lung function examination; or oral or facial pain aggravation when chewing. Subjects who had mental illness and cognitive disorders were also excluded.

### Study design

**Figure 1:**
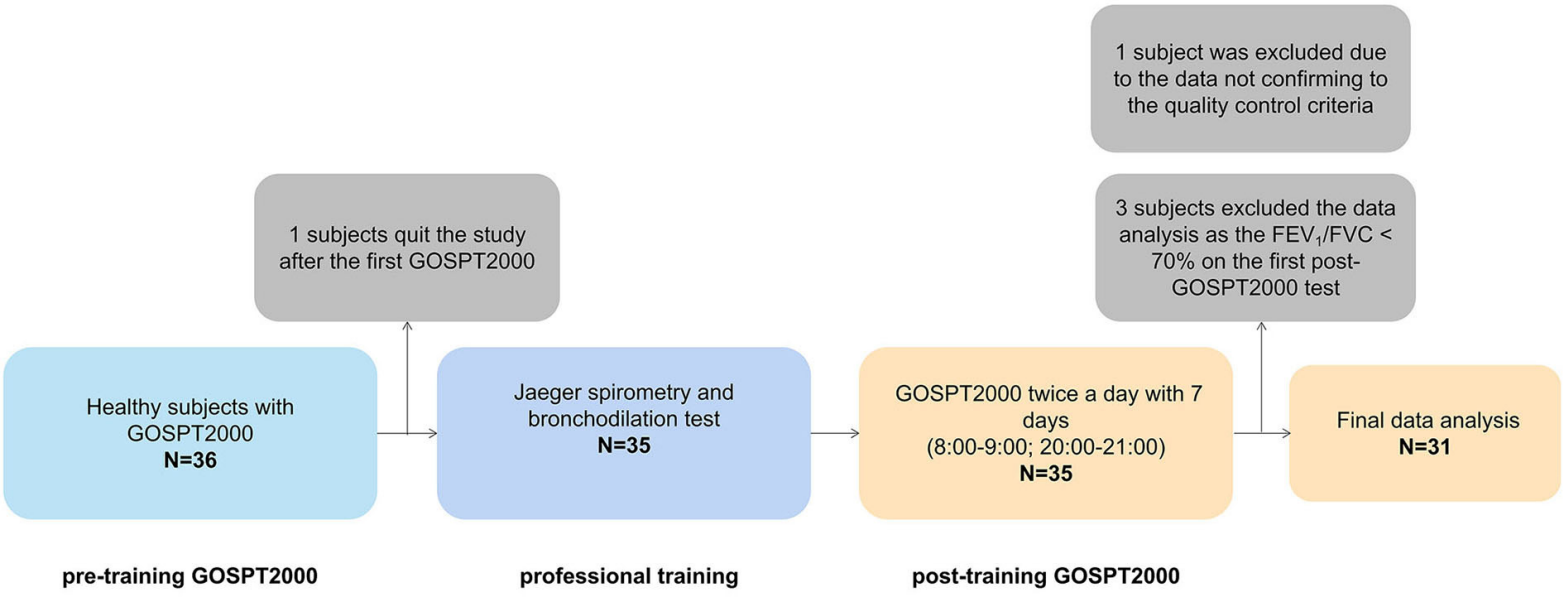
Schematic presentation of study design. A total of 36 adults were invited to participate, among which one participant quit the study after conducted the first GOSPT2000 test and 35 adults attended and completed the 7-day portable spirometer and laboratory pulmonary function tests; of these, three participants were excluded from the data analysis as their FEV_1_/FVC was < 70% on the first GOSPT2000 test, and one person was excluded from final analysis as data did not meet the quality control criteria, leaving 31 participants for the final data analysis in the present study. After the first portable GOSPT2000 test, the subjects were asked to complete the spirometry and bronchodilation test using a Jaeger spirometer under the guidance of a trained medical technician on the same morning. The quiescent period between measurements was 20 mins after completing the spirometry and bronchodilation test. Then, the subjects took the GOSPT2000 device home and completed the pulmonary function monitoring in the home setting for the next 7 consecutive days. FVC, forced vital capacity; FEV_1_, forced expiratory volume in 1 s; FEV_3_, FEV in 3 s; MEF50: forced expiratory flow at 50% of forced vital capacity; MEF25, forced expiratory flow at 75% of forced vital capacity; MMEF, forced expiratory flow between 25 and 75%; PEF, peak expiratory flow; SD, standard deviation; CI, confidence interval; t-, total; m-, morning; e-, evening.

A schematic representation of our study is presented in Figure 1. After providing written informed consent, participant demographics, occupation, education, height, weight, smoking status, and current medical status (including acute illnesses in the previous 4 weeks) were assessed. After reconfirming subjects were free of respiratory symptoms, we asked each participant to take a portable GOSPT2000 test relying only on video teaching. No guidance on the detection method of the GOSPT2000 equipment was given by trained medical technicians during the measurement.

After the first pre-training portable GOSPT2000 test, the subjects were asked to complete spirometry and a bronchodilation test using a hospital spirometer (Jaeger Co., Hoechberg, Germany) under the guidance of a trained medical technician on the same morning. The quiescent period was over 20 min before the spirometry and bronchodilation test. Then, the subjects took the GOSPT2000 device home and completed the pulmonary function monitoring in the home setting for the next 7 consecutive days. The detection time followed the setting of the software system and was carried out between 08:00 and 09:00 in the morning and between 20:00 and 21:00 in the evening. The GOSPT2000 equipment was returned after 7 consecutive days of measurements.

### Portable spirometer (GOSPT2000)

GOSPT2000, a product of GoSprio (Monitored Therapeutics Inc., Dublin, OH, USA) has been approved by the FDA in the United States (K163249).

The GOSPT2000 portable spirometer is a small, handheld device consisting of a vertical turbine volume sensor. The turbine transducer measures expired air directly at body temperature and pressure with saturated water vapor (BTPS), thus avoiding inaccuracies in temperature corrections. The GOSPT2000 performs full flow–volume loops, including inspiration and expiration data. It has built-in quality control measurements and transmits indices of measurement quality including time to peak flow, BEV, total expiratory time, end-expiratory flow detection, and identification of cough during the measurement. It transmits real-time lung function data to computers, tablets, or smartphones over a Bluetooth connection for telehealth applications.

Measurements were performed following the detailed and standardized operation video after subjects responded to a symptoms questionnaire. Forced expiratory maneuvers that met all acceptability criteria were performed until the two best efforts were reproducible (minimum of three). The test curve with the highest sum of the FVC and FEV_1_ was considered the best one, and the largest FVC and FEV_1_ measurements were recorded. Between each set of measurements, the subjects rested 5–10 mins.

Data on PEF, FVC, FEV_1_, FEV_3_, forced expiratory flow at 50% of forced vital capacity (MEF50), forced expiratory flow at 75% of forced vital capacity (MEF25), and forced expiratory flow between 25 and 75% (MMEF) were collected for analysis. According to the latest standardization of spirometry in the 2019 update (24), the back-extrapolated volume (BEV) must be 5% of the FVC or 0.100 L to ensure that FEV_1_ results from a maximal effort.

Diurnal variation was calculated from twice-daily spirometry variables as highest value of the day minus lowest value of the day/mean of highest and lowest values of the day, averaged for 1 week.

### Spirometry and bronchodilation test

Salbutamol (400 ug) with a metered dose inhaler was used as a bronchodilator in the bronchodilation test, and spirometry was performed before and 20 min after bronchodilator use. The improvement of the FEV_1_ was calculated and expressed as percentage changes compared with the baseline values.

Spirometry was performed by using a spirometer (Jaeger Co., Hoechberg, Germany) following the performance criteria recommended in the ATS/ERS Standardization of Spirometry (9). The following parameters were collected: PEF, FVC, FEV_1_, FEV_3_, MEF50, MEF25, and MMEF expressed as absolute value. FEV_1_/FVC, FEV_3_/FVC, MEF50/FVC, MEF25/FVC, and MMEF/FVC were the ratio of two parameters.

PEF, FEV_1_, FEV_1_/FVC, and MEF75 represent large-airway function; MEF50, MEF25, and MMEF represent small-airway function.

### Statistical analysis

Analyses were performed by GraphPad Prism version 9.01 (GraphPad Software, San Diego, CA, USA), SPSS version 20.0 (SPSS Inc, Chicago, Illinois, USA), and R version 4.1.1 (Innovative Solutions, St. Louis, MO, USA).

Baseline data are presented descriptively. Normality of distribution was checked using the Shapiro–Wilk test. Normally distributed data were expressed as mean ± standard deviation (SD) or 95% confidence interval (CI). Non-normally distributed data were expressed as median and interquartile ranges (IQR). The coefficient of variation (CV) and diurnal variation were calculated for each continuous variable.

A Mann–Whitney U test was performed to compare inter-group differences between two medians of categorical variables (gender). A Kruskal–Wallis test was performed to compare inter-group differences between four medians of categorical variables (age and education degree). The Spearman correlation coefficient matrix and Spearman rank correlation tests were performed by R version 4.1.1. The correlation between different variables was determined using Spearman analysis; *r* > 0.4 or < -0.4 was defined as strong correlation, and *r* between −0.4 and 0.4 as weak correlation, if *P* < 0.05. Splines of large- and small-airway function with age were smoothed using the LOESS method. Simple linear regression was also performed by GraphPad Prism version 9.01. The threshold for statistical significance for all analyses was set at *P* < 0.05.

## Results

### Demographic and baseline characteristics

Of the 36 healthy adults invited to participate, one participant quit the study after the first pre-training GOSPT2000 test; thereafter, 35 adults attended and completed the laboratory pulmonary function tests and the spirometry test for next 7 days morning and evening using the portable spirometer GOSPT2000. A total of three participants were excluded from data analysis because the FEV_1_/FVC was < 70% on the first post-training GOSPT2000 spirometry test, and one more was excluded because the BEV was >5% and >0.100 L of the FVC according to the built-in quality control of GOSPT2000 device, leaving 31 participants for final data analysis (Figure 1). No subject quit the home monitoring during the 7 days. Of the 448 sets of spirometry data, only 16 sets (10 sets from one person excluded for final analysis; four sets from another person excluded for analysis of diurnal variation) did not conform to the acceptability and repeatability criteria. All the participants had normal Jaeger spirometer measurement results and negative bronchodilation tests with improvement of FEV_1_ < 200 ml and FEV_1_/FVC < 20%.

The mean age of subjects analyzed was 36.68 (SD 11.64) years, and 58.06% (18 of 31) were female. The average height of the subjects was 168.9 (SD 8.791) cm, the average body weight was 65.55 (SD 10.38) kg, and the average BMI was 22.95 (SD 2.950) kg/m^2^ (Table 1). Overall, 29.03% (9 of 31) of the study population graduated from junior high school, 35.48% (11 of 31) graduated from senior high school, and 35.48% (11 of 31) had a university-level education. All their home-monitoring pulmonary function measurement variable (FVC, FEV_1_, FEV_3_, PEF, MEF50, MEF25, MMEF, FEV_1_/FVC, FEV_3_/FVC, MEF50/FVC, MEF25/FVC, and MMEF/FVC) descriptive data are presented in Table 1.

**Table 1:** Demographic data and portable spirometry variable values of non-smoking healthy adults.

**Variables**	**25% Percentile**	**Median, %**	**75% Percentile**	**Mean**	**SD**	**Lower 95% CI** **of mean**	**Upper 95% CI of mean**	***P*** **^b^**	***df***
Age, years	24	37	46	36.68	11.64	32.41	40.95	0.1338	30
Height, cm	163	167	177	168.9	8.791	165.7	172.1	0.811	30
Weight, kg	60	65	72	65.55	10.38	61.74	69.36	0.3095	30
BMI, kg/m^2^	20.2	23.24	24.34	22.95	2.95	21.86	24.03	0.1727	30
tFVC	3.189	3.622	4.504	3.853	0.8661	3.535	4.171	0.2367	30
tFEV_1_	2.621	2.929	3.651	3.147	0.7721	2.864	3.431	0.1477	30
tFEV_1_/FVC	0.773	0.8193	0.8548	0.8154	0.05681	0.79.45	0.8362	0.5599	30
tFEV_3_	3.058	3.462	4.390	3.715	0.8817	3.392	4.038	0.1802	30
**tFEV**_**3**_ **/FVC**	**0.9458**	**0.9676**	**0.9815**	0.9620	0.0287	0.9515	0.9725	**0.0146**	30
**tPEF^a^**	**6.502**	**7.181**	**9.378**	7.978	1.816	7.311	8.644	**0.0113**	29
tMEF50	2.960	3.841	4.219	3.700	1.063	3.311	4.090	0.3475	30
tMEF50/FVC	0.8188	0.9982	1.097	0.9680	0.2106	0.8907	1.045	0.7233	30
**tMEF25**	**1.001**	**1.250**	**1.762**	0.7038	1.085	1.213	1.729	**0.0125**	30
tMEF25/FVC	0.2826	0.3667	0.4497	0.3735	0.1305	0.3256	0.4213	0.1211	30
tMMEF	2.522	3.174	3.742	3.267	1.085	2.869	3.665	0.4763	30
tMMEF/FVC	0.6790	0.8683	0.9874	0.8471	0.2013	0.7733	0.9210	0.7791	30
mFVC	3.208	3.666	4.573	3.823	0.8907	3.497	4.15	0.1731	30
eFVC	3.161	3.543	4.434	3.765	0.9028	3.434	4.096	>0.1	30
mFEV_1_	2.627	2.988	3.654	3.141	0.7934	2.85	3.432	0.1668	30
eFEV_1_	2.577	2.876	3.653	3.096	0.8074	2.8	3.392	0.0946	30
mFEV_3_	3.07	3.487	4.45	3.692	0.9134	3.357	4.027	0.1598	30
eFEV_3_	3.001	3.42	4.33	3.639	0.9233	3.3	3.977	>0.1	30
mFEV_1_/FVC	0.7825	0.8217	0.8587	0.8199	0.05072	0.8013	0.8385	0.9448	30
eFEV_1_/FVC	0.7894	0.8232	0.8551	0.8201	0.04953	0.8019	0.8382	0.9241	30
mFEV_3_/FVC	0.9416	0.9651	0.9874	0.9633	0.02795	0.953	0.9735	0.054	30
eFEV_3_/FVC	0.9463	0.9682	0.9821	0.9638	0.02576	0.9544	0.9733	0.0869	30
**mPEF^a^**	**6.311**	**7.093**	**9.149**	7.796	1.851	7.117	8.475	**0.0231**	29
**ePEF^a^**	**6.457**	**7.157**	**9.527**	7.939	1.914	7.237	8.641	**0.0001**	29
mMEF50	2.946	3.851	4.184	3.691	1.039	3.309	4.072	0.3922	30
eMEF50	3.034	3.733	4.184	3.717	1.079	3.321	4.113	>0.1	30
**mMEF25**	**1.007**	**1.297**	**1.793**	1.489	0.7069	1.229	1.748	**0.0199**	30
eMEF25	1.003	1.253	1.761	1.458	0.7018	1.201	1.715	>0.1	30
mMMEF	2.639	3.23	3.7	3.279	1.081	2.883	3.676	0.3559	30
eMMEF	2.633	3.197	3.784	3.268	1.078	2.873	3.664	>0.1	30
mMEF50/FVC	0.8175	0.9866	1.086	0.9734	0.193	0.9025	1.044	0.5711	30
eMEF50/FVC	0.8794	0.9833	1.112	0.9945	0.1996	0.9213	1.068	0.4387	30
mMEF25/FVC	0.2737	0.3589	0.4542	0.3806	0.1282	0.3336	0.4276	0.0995	30
**eMEF25/FVC**	**0.2895**	**0.3795**	**0.4354**	0.3769	0.1243	0.3313	0.4225	**0.0189**	30
mMMEF/FVC	0.7007	0.8925	0.9737	0.8565	0.1868	0.788	0.9251	0.8277	30
eMMEF/FVC	0.718	0.8717	0.9978	0.8659	0.1848	0.7982	0.9337	0.9922	30

df, degrees of freedom; FVC, forced vital capacity; FEV_1_, forced expiratory volume in 1 s; FEV_3_, FEV in 3 s; MEF50: forced expiratory flow at 50% of forced vital capacity; MEF25: forced expiratory flow at 75% of forced vital capacity; MMEF: forced expiratory flow between 25 and 75%; PEF, peak expiratory flow; SD, standard deviation; CI, confidence interval; t-, total; m-, morning; e-, evening.

a n = 30.

b P-values in this table show whether the data conform to a normal distribution. P < 0.05 indicates that the data conform to a normal distribution; otherwise, the data conform to non-normal distribution.

Bold values indicates statistical significance.

### Factors associated with large- and small-airway function both in the morning and evening for 7 consecutive days

**Figure 2:**
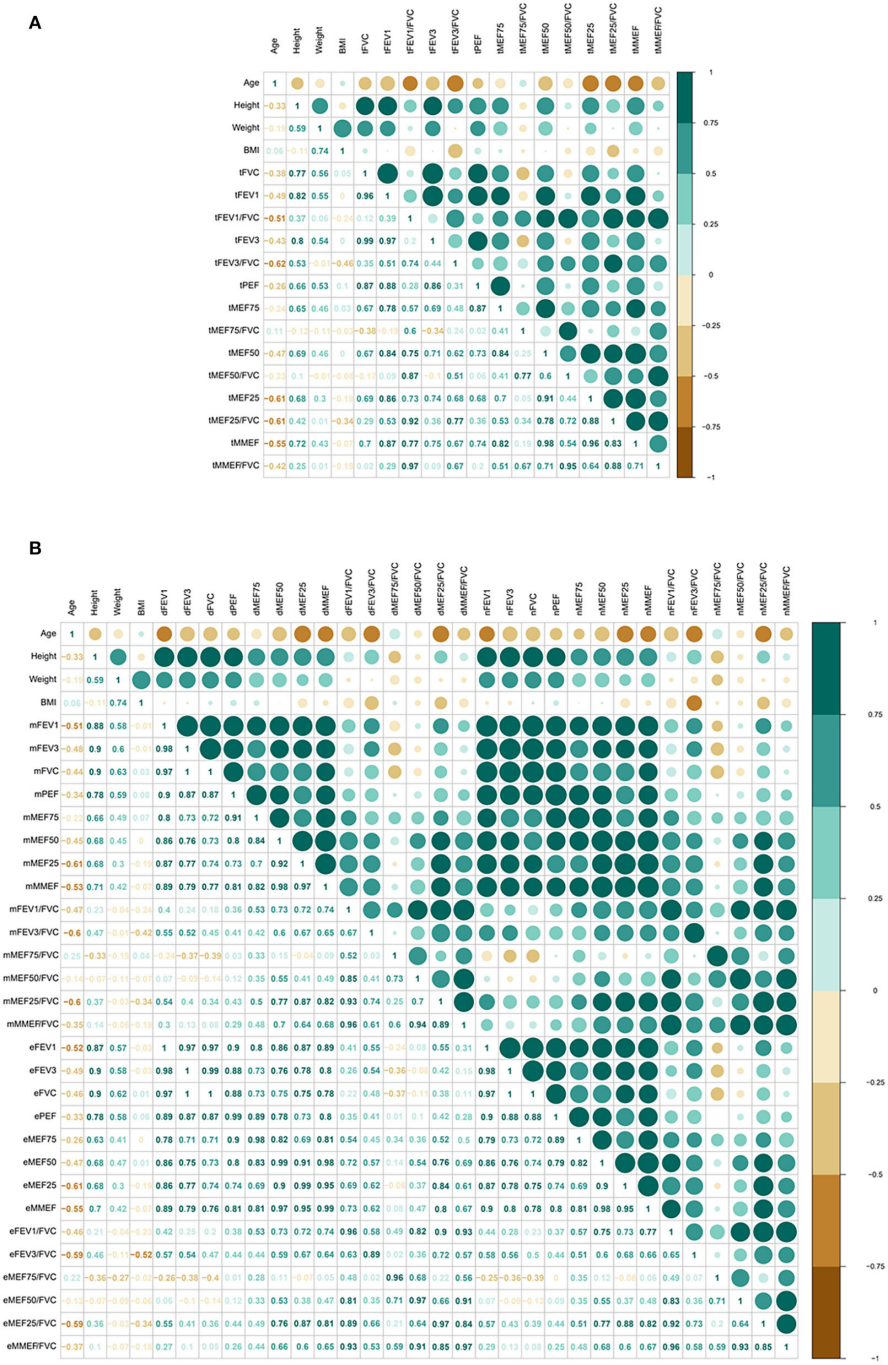
Correlation of pulmonary function (total, morning, and evening) with age, height, weight, and BMI (*N* = 31, except *N* = 30 for PEF). Both large- and small-airway function variable values were strongly negatively related to age, and were dramatically positively related to height **(A)**. Similar correlation both in the morning and the evening was found **(B)**. FVC, forced vital capacity; FEV_1_, forced expiratory volume in 1 s; FEV_3_, FEV in 3 s; MEF50: forced expiratory flow at 50% of forced vital capacity; MEF25: forced expiratory flow at 75% of forced vital capacity; MMEF: forced expiratory flow between 25 and 75%; PEF, peak expiratory flow; SD, standard deviation; CI, confidence interval; t-, total; m-, morning; e-, evening.

The Spearman correlation coefficient matrix and Spearman rank correlation tests were performed (Figure 2A; Supplementary Table 1). Both large- and small-airway function variable values were strongly negatively related to age (*P* ≤ 0.0001 for tMEF25 and tMEF25/FVC; *P* < 0.001 for tFEV_3_/FVC; *P* < 0.01 for tFEV_1_, tFEV_1_/FVC, tMEF50, and tMMEF; and *P* < 0.05 for tFEV_3_ and tMMEF/FVC) and were dramatically positively related to height (*P* < 0.0001 for PEF, tFVC, tFEV_1_, tFEV_3_, tMEF50, tMEF25, and tMMEF; *P* < 0.05 for tFEV_3_/FVC and tMEF25/FVC). No significant correlations between age and FVC, PEF, or MEF50/FVC, and no significant correlations between height and FEV_1_/FVC, MEF50/FVC, or MMEF/FVC were found (*P* > 0.05). The MEF/FVC ratio, which reflects effort-independent expiratory airflow in the context of lung volume, reflected less correlation with age, height, and weight, especially with the height and weight. Both large- and small-airway function absolute values of male adults were dramatically higher than those of female adults (Supplementary Figure 1); however, FEV_1_/FVC (Supplementary Figure 1E) and MEFs/FVC (Supplementary Figures 1J–L) showed no difference across sex (*P* > 0.05).

Then, we further compared the morning and evening variable values of large- and small-airway function, separately, and similar correlation in the morning and in the evening was found (Figure 2B; Supplementary Table 1).

### Age-related features in large- and small-airway function in non-smoking healthy adults

**Figure 3:**
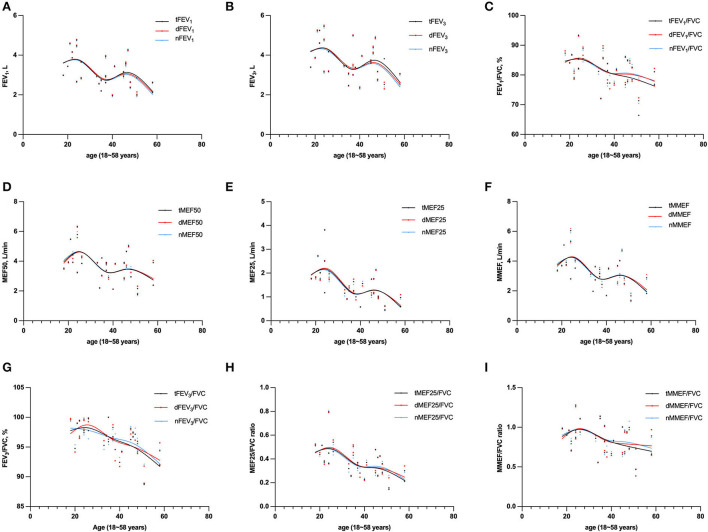
Absolute values for FEV_1_ **(A)**, FEV_3_ **(B)**, MEF50 **(D)**, MEF25 **(E)**, and MMEF **(F)**, ratio values for FEV_1_/FVC **(C)**, FEV_3_/FVC **(G)**, MEF25/FVC **(H)**, and MMEF/FVC **(I)** by age (*N* = 31, except *N* = 30 for PEF). Graphs were generated to illustrate characteristics of large- and small airway function values by age. FVC, forced vital capacity; FEV_1_, forced expiratory volume in 1 s; FEV_3_, FEV in 3 s; MEF50: forced expiratory flow at 50% of forced vital capacity; MEF25: forced expiratory flow at 75% of forced vital capacity; MMEF: forced expiratory flow between 25 and 75%; PEF, peak expiratory flow; SD, standard deviation; CI, confidence interval; t-, total; m-, morning; e-, evening.

A smoothing age spline was generated to illustrate characteristics of large- and small-airway function values by age (Figure 3). A small increasing trend of both large- and small-airway function from 18 years of age to 25 years of age was found. There was then a drop in the trend until about 35 years of age, followed by a small but steady increase till 50 years of age, followed by a rapid decline in both large- and small-airway function till old age (Figure 3).

All their 7-day GOSPT2000 pulmonary function measurement variable descriptive data grouped by age are presented in Table 2. There were no difference in sex, height, weight, and BMI among the groups across age. In the 30- to 45-year and 45- to 60-year subgroups, both large- (*P* = 0.047 for FVC; *P* = 0.007 for FEV_1_; and *P* = 0.019 for FEV_1_/FVC) and small-airway function variable (*P* = 0.014 for MEF50; *P* < 0.001 for MEF25; and *P* = 0.004 for MMEF) values were lower than those in the 18- to 30-year subgroup. For the tMEF/FVC ratio, tMEF25/FVC values in the 18- to 30-year subgroup were higher than those in the other two subgroups. No significant age-related differences were found between the subgroups for PEF (*P* > 0.05).

**Table 2:** Portable spirometry variable values according to age.

**Variables**	**Age, years**				***P***
	**18–30**	**30–45**	**45–60**	**All subjects**	
*N*	10	10	11	31	–
Height, m	1.734 (0.0757)	1.660 (0.0908)	1.675 (0.0866)	1.689 (0.0879)	0.134
Weight, kg	68.7 (12.54)	65.6 (11.94)	62.64 (5.853)	65.55 (10.38)	0.423
BMI, kg/m^2^	22.76 (3.256)	23.71 (3.369)	22.41 (2.328)	22.95 (2.950)	0.600
Sex (female)	4/10 (40%)	7/10 (70%)	7/11 (63.64%)	18/31 (58.06%)	>0.05
**tFVC, L**	**4.3872 (0.8870)**	**3.4918 (0.7638)**	**3.696 (0.7578)**	**3.853 (0.8661)**	**0.047**
**tFEV**_**1**_ **, L**	**3.742 (0.7549)**	**2.805 (0.5665)**	**2.919 (0.6775)**	**3.147 (0.7722)**	**0.007**
**tFEV**_**3**_ **, L**	**4.307 (0.8777)**	**3.3556 (0.7528)**	**3.5032 (0.7742)**	**3.715 (0.8817)**	**0.027**
tPEF^*^, L/min	8.9687 (1.806)	7.1167 (1.165)	7.8593 (1.999)	7.9776 (1.816)	0.067
**tMEF50**, L/min	**4.4752 (1.032)**	**3.3325 (0.7384)**	**3.3301 (1.025)**	**3.700 (1.062)**	**0.014**
**tMEF25**, L/min	**2.123 (0.7311)**	**1.1857 (0.3112)**	**1.137 (0.5374)**	**1.471 (0.7038)**	**< 0.001**
**tMMEF**, L/min	**4.155 (1.004)**	**2.8673 (0.6568)**	**2.8226 (1.036)**	**3.267 (1.085)**	**0.004**
**tFEV**_**1**_ **/FVC**	**0.8539 (0.0408)**	**0.8069 (0.049)**	**0.7880 (0.0603)**	**0.8154 (0.0568)**	**0.019**
**tFEV**_**3**_ **/FVC**	**0.9818 (0.0163)**	**0.9605 (0.0213)**	**0.9453 (0.0334)**	**0.9620 (0.0287)**	**0.009**
tMEF50/FVC	1.027 (0.1542)	0.9763 (0.2355)	0.9065 (0.2325)	0.968 (0.2106)	0.432
**tMEF25/FVC**	**0.4831 (0.1269)**	**0.3430 (0.0825)**	**0.3015 (0.1088)**	**0.968 (0.2106)**	**0.002**
tMMEF/FVC	0.9523 (0.1522)	0.8370 (0.1990)	0.7607 (0.2137)	0.8471 (0.2013)	0.088
tFVC%	93.19 (6.760)	92.7023 (10.33)	106.8 (26.93)	97.86 (18.24)	0.128
tFEV_1_%	93.02 (7.753)	87.3655 (7.399)	99.30 (18.52)	93.42 (13.18)	0.114
tPEF%	100.5 (11.71)	95.83 (10.01)	108.7 (21.47)^*^	101.9 (15.96)	0.173
tMEF50%	85.29 (14.01)	74.1309 (15.58)	78.0096 (19.18)	79.11 (16.61)	0.322
**tMEF25%**	**84.58 (24.77)**	**58.21 (11.50)**	**64.18 (23.21)**	**68.83 (23.06)**	**0.021**
**t**MMEF%	87.77 (15.58)	72.54 (14.70)	78.52 (22.90)	79.58 (18.76)	0.19
**mFVC, L**	**4.403 (0.9005)**	**3.523 (0.7583)**	**3.574 (0.7947)**	**3.825 (0.7947)**	**0.038**
**eFVC, L**	**4.371 (0.8750)**	**3.463 (0.7722)**	**3.490 (0.8208)**	**3.766 (0.9021)**	**0.03**
**mFEV**_**1**_ **, L**	**3.755 (0.7583)**	**2.828 (0.5696)**	**2.868 (0.7213)**	**3.141 (0.7930)**	**0.007**
**eFEV**_**1**_ **, L**	**3.728 (0.7523)**	**2.784 (0.5652)**	**2.807 (0.7539)**	**3.097 (0.8071)**	**0.007**
**mFEV**_**3**_ **, L**	**4.329 (0.8903)**	**3.38 (0.7528)**	**3.398 (0.8250)**	**3.693 (0.9132)**	**0.022**
**eFEV**_**3**_ **, L**	**4.286 (0.8663)**	**3.333 (0.7540)**	**3.331 (0.8666)**	**3.639 (0.9228)**	**0.021**
**mFEV**_**1**_ **/FVC**	**0.8545 (0.0432)**	**0.8071 (0.0525)**	**0.8002 (0.0421)**	**0.8199 (0.0507)**	**0.025**
**eFEV**_**1**_ **/FVC**	**0.8539 (0.0393)**	**0.8079 (0.0464)**	**0.8004 (0.0481)**	**0.8201 (0.0495)**	**0.024**
**mFEV**_**3**_ **/FVC**	**0.9833 (0.0183)**	**0.9596 (0.0253)**	**0.9484 (0.0284)**	**0.9633 (0.0280)**	**0.01**
**eFEV**_**3**_ **/FVC**	**0.9805 (0.0157)**	**0.9625 (0.0195)**	**0.9498 (0.0305)**	**0.9638 (0.0257)**	**0.018**
mPEF, L/min	8.904 (1.777)	7.031 (1.086)	7.486 (2.111)^*^	7.796 (1.851)	0.056
ePEF, L/min	9.033 (1.839)	7.212 (1.251)	7.606 (2.164)^*^	7.939 (1.913)	0.076
**mMEF50**, L/min	**4.431 (1.017)**	**3.323 (0.7548)**	**3.332 (0.9809)**	**3.684 (1.037)**	**0.016**
**eMEF50**, L/min	**4.519 (1.056)**	**3.318 (0.7141)**	**3.356 (1.043)**	**3.719 (1.080)**	**0.012**
**mMEF25**, L/min	**2.143 (0.7298)**	**1.203 (0.3385)**	**1.147 (0.5327)**	**1.486 (0.7082)**	**< 0.001**
**eMEF25**, L/min	**2.103 (0.7346)**	**1.175 (0.3039)**	**1.130 (0.5425)**	**1.458 (0.7017)**	**0.001**
**mMMEF**, L/min	**4.149 (1.040)**	**2.870 (0.6922)**	**2.848 (0.9908)**	**3.275 (1.082)**	**0.004**
**eMMEF**, L/min	**4.160 (0.9730)**	**2.861 (0.6321)**	**2.830 (1.049)**	**3.269 (1.078)**	**0.003**
mMEF50/FVC	1.015 (0.1573)	0.9668 (0.2383)	0.9414 (0.1884)	0.9734 (0.1930)	0.691
eMEF50/FVC	1.041 (0.1550)	0.9822 (0.2321)	0.9638 (0.2146)	0.9945 (0.1996)	0.674
**mMEF25/FVC**	**0.4874 (0.1280)**	**0.3467 (0.0914)**	**0.3143 (0.0981)**	**0.3806 (0.1282)**	**0.002**
**eMEF25/FVC**	**0.4806 (0.1283)**	**0.3428 (0.0775)**	**0.3136 (0.0991)**	**0.3769 (0.1243)**	**0.002**
mMMEF/FVC	0.9483 (0.1620)	0.8325 (0.2048)	0.7950 (0.1737)	0.8565 (0.1868)	0.153
eMMEF/FVC	0.9581 (0.1444)	0.8442 (0.1952)	0.8020 (0.1896)	0.8659 (0.1848)	0.139

FVC, forced vital capacity; FEV_1_, forced expiratory volume in 1 s; FEV_3_, FEV in 3 s; MEF50: forced expiratory flow at 50% of forced vital capacity; MEF25: forced expiratory flow at 75% of forced vital capacity; MMEF: forced expiratory flow between 25 and 75%; PEF, peak expiratory flow; SD, standard deviation; CI, confidence interval; t-, total; m-, morning; e-, evening.

* n = 10.

Bold values indicates statistical significance.

We further compared the morning and evening variable values of large- and small-airway function, separately. We found a similar phenomenon both in the morning and the evening, and non-smoking healthy adults ≥30 years had lower large- and small-airway function values than adults <30 years (Table 2).

### The variation features of large- and small-airway function in non-smoking healthy adults were also correlated with age

**Figure 4:**
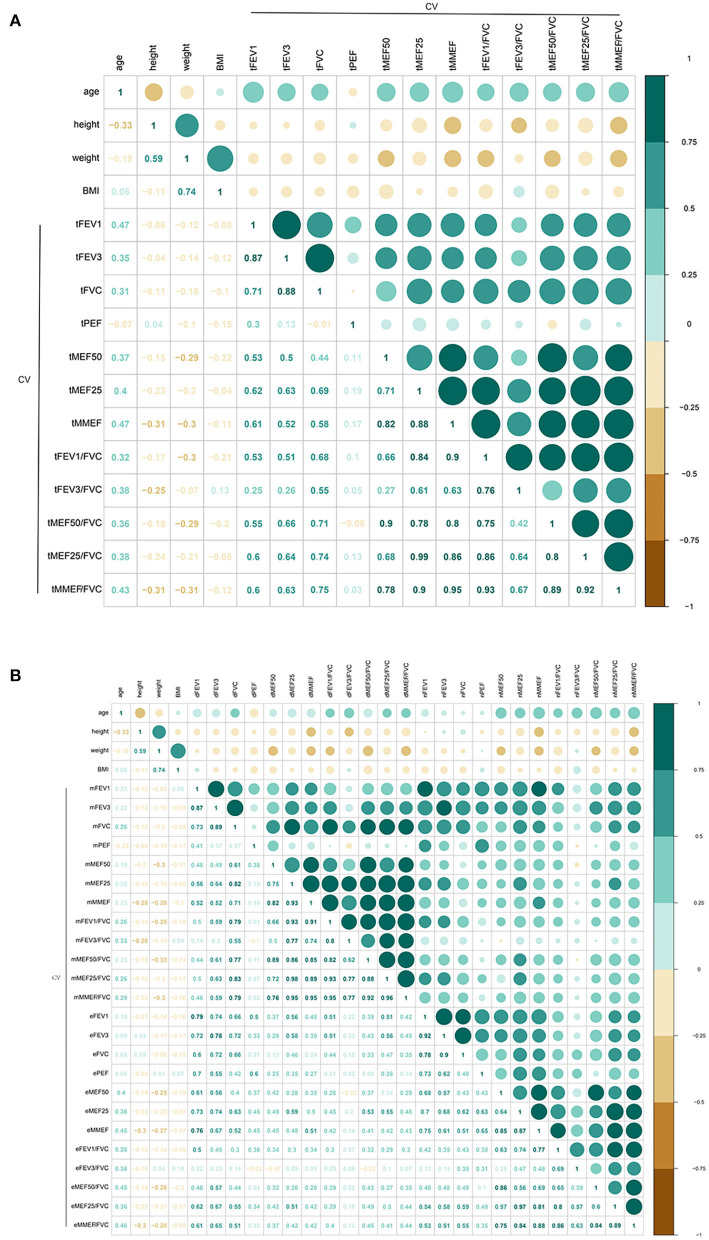
Correlation of CV of pulmonary function variable values with age, height, weight, and BMI (total, morning, and evening) (*N* = 31, except *N* = 30 for PEF). A strong relationship between age and both large- [**(A)**, *r* = 0.47, *P* = 0.0082 for tFEV_1_] and small-airway (tMEF25, tMMEF, and tMMEF/FVC) function variables [**(A)**, *r* ≥ 0.4, *P* < 0.05 for all]. tMEF50, tFEV_3_/FVC, tMEF50/FVC, and tMEF25/FVC was weekly correlated to age [**(A)**, 0.36 ≤ *r* ≥ 0.38, *P* < 0.05 for all], no significant relationships between height, weight, or BMI and both large- and small-airway function variables were found [**(A)**, *P* > 0.05]. Similar correlation both in the morning and the evening was found **(B)**. FVC, forced vital capacity; FEV_1_, forced expiratory volume in 1 s; FEV_3_, FEV in 3 s; MEF50, forced expiratory flow at 50% of forced vital capacity; MEF25, forced expiratory flow at 75% of forced vital capacity; MMEF, forced expiratory flow between 25 and 75%; PEF, peak expiratory flow; SD, standard deviation; CI, confidence interval; t-, total; m-, morning; e-, evening.

We then analyzed the relationships between the CV of lung function variables and age, height, weight, or BMI. A strong relationship between age and both large- (*r* = 0.47, *P* = 0.0082 for tFEV_1_) and small-airway (tMEF25, tMMEF, and tMMEF/FVC) function variables was found (Figure 4; Supplementary Table 2, *r* ≥ 0.4, *P* < 0.05 for all). tMEF50, tFEV_3_/FVC, tMEF50/FVC, and tMEF25/FVC were weekly correlated to age (0.36 ≤ *r* ≥ 0.38, *P* < 0.05 for all); no significant relationships between height, weight, or BMI and both large- and small-airway function variables were found (*P* > 0.05). There was also no significant difference across sex (except *P* < 0.05 for MMEF/FVC only, *P* > 0.05 for all other variables, Supplementary Figure 2).

To validate the influence of age on the CV of lung function variables, we compared the CV of lung function variables in the three subgroups across age. Of the values of large-airway function, the CV was significantly higher in the 45- to 60-year subgroup (4.666 ± 1.946, *P* = 0.002 for FEV_1_; 4.565 ± 2.478, *P* = 0.017 for FEV_3_, Table 3) than in the 30- to 45-year subgroup (2.906 ± 0.8121 for FEV_1_; 2.739 ± 0.8813 for FEV_3_) and the 18- to 30-year subgroup (2.489 ± 0.8739 for FEV_1_; 2.648 ± 0.8069 for FEV_3_).

**Table 3:** Comparison of the CV of large- and small-airway function variables according to age.

**CV of variables, %**	**Age, years**				***P***
	**18~30**	**30~45**	**45~60**	**All subjects**	
*N*	10	10	11	31	–
tFVC	2.821 (0.7225)	3.572 (1.668)	4.712 (2.562)	3.734 (1.954)	0.078
**tFEV**_**1**_	**2.489 (0.8739)**	**2.906 (0.8121)**	**4.666 (1.946)**	**3.396 (1.623)**	**0.002**
**tFEV**_**3**_	**2.648 (0.8069)**	**2.739 (0.8813)**	**4.565 (2.478)**	**3.357 (1.818)**	**0.017**
tPEF	4.846 (1.698)	4.588 (1.335)	4.496 (1.937)^*^	4.639 (1.635)	0.888
**tMEF50**	**7.99 (1.951)**	**7.385 (2.392)**	**10.38 (3.196)**	**8.641 (2.834)**	**0.031**
tMEF25	10.59 (4.202)	14.44 (7.357)	17.82 (8.521)	14.40 (7.406)	0.078
**tMMEF**	**6.603 (1.752)**	**7.951 (4.535)**	**11.21 (4.178)**	**8.674 (4.108)**	**0.023**
mFVC	2.289 (1.185)	3.562 (1.882)	4.248 (3.352)	3.395 (2.432)	0.1790
eFVC	3.009 (0.9634)	3.098 (1.689)	3.856 (2.306)	3.338 (1.750)	0.4861
mFEV_1_	2.386 (1.381)	2.455 (0.7676)	3.744 (2.145)	2.89 (1.642)	0.0960
eFEV_1_	2.413 (0.7666)	2.939 (1.328)	3.866 (2.218)	3.098 (1.651)	0.122
mFEV_3_	2.348 (1.178)	2.414 (0.7558)	4.058 (3.349)	2.976 (2.234)	0.1350
eFEV_3_	2.643 (1.021)	2.691 (1.323)	3.533 (2.356)	2.974 (1.693)	0.408
mPEF	5.209 (2.465)	3.752 (1.520)	3.829 (2.589)^*^	4.249 (2.281)	0.2790
ePEF	4.173 (1.777)	4.043 (1.512)	4.686 (1.859)^*^	4.313 (1.693)	0.667
mMEF50	8.054 (3.132)	7.837 (3.926)	9.516 (3.687)	8.502 (3.562)	0.5130
**eMEF50**	**7.291 (2.855)**	**6.359 (2.655)**	**11.18 (3.427)**	**8.371 (3.621)**	**0.002**
mMEF25	9.919 (3.911)	15.44 (11.91)	16.38 (8.862)	13.99 (9.036)	0.2220
**eMEF25**	**11.18 (5.631)**	**11.05 (3.809)**	**19.04 (10.59)**	**13.93 (8.133)**	**0.028**
mMMEF	6.586 (2.288)	8.612 (7.248)	10.18 (5.087)	8.516 (5.312)	0.3100
**eMMEF**	**6.43 (2.122)**	**6.459 (2.286)**	**11.90 (4.640)**	**8.380 (4.139)**	**0.001**

FVC, forced vital capacity; FEV_1_, forced expiratory volume in 1 s; FEV_3_, FEV in 3 s; MEF50: forced expiratory flow at 50% of forced vital capacity; MEF25, forced expiratory flow at 75% of forced vital capacity; MMEF, forced expiratory flow between 25 and 75%; PEF, peak expiratory flow; SD, standard deviation; CI, confidence interval; t-, total; m-, morning; e-, evening.

* n = 10.

Bold values indicates statistical significance.

For MEFs, the 45- to 60-year subgroup showed higher CV values of MEF50 (10.38 ± 3.196, *P* = 0.031) and MMEF (11.21 ± 4.178, *P* = 0.023) than the 30- to 45-year subgroup (7.385 ± 2.392 for MEF50; 7.951 ± 4.535 for MMEF) and 18- to 30-year subgroup (7.990 ± 1.951 for MEF50; 6.603 ± 1.752 for MMEF, Table 3).

When circadian rhythms were considered, only the evening CV values of small-airway function variables were higher in the 45- to 60-year subgroup (11.18 ± 3.427, *P* = 0.002 for MEF50; 19.04 ± 10.59, *P* = 0.028 for MEF25; 11.90 ± 4.640, *P* = 0.001 for MMEF, Table 3), while there were no age-related differences for large-airway function variables (*P* > 0.05 for FVC, FEV_1_, and FEV_3_, Table 3) and for morning variable values (*P* > 0.05 for all).

**Figure 5:**
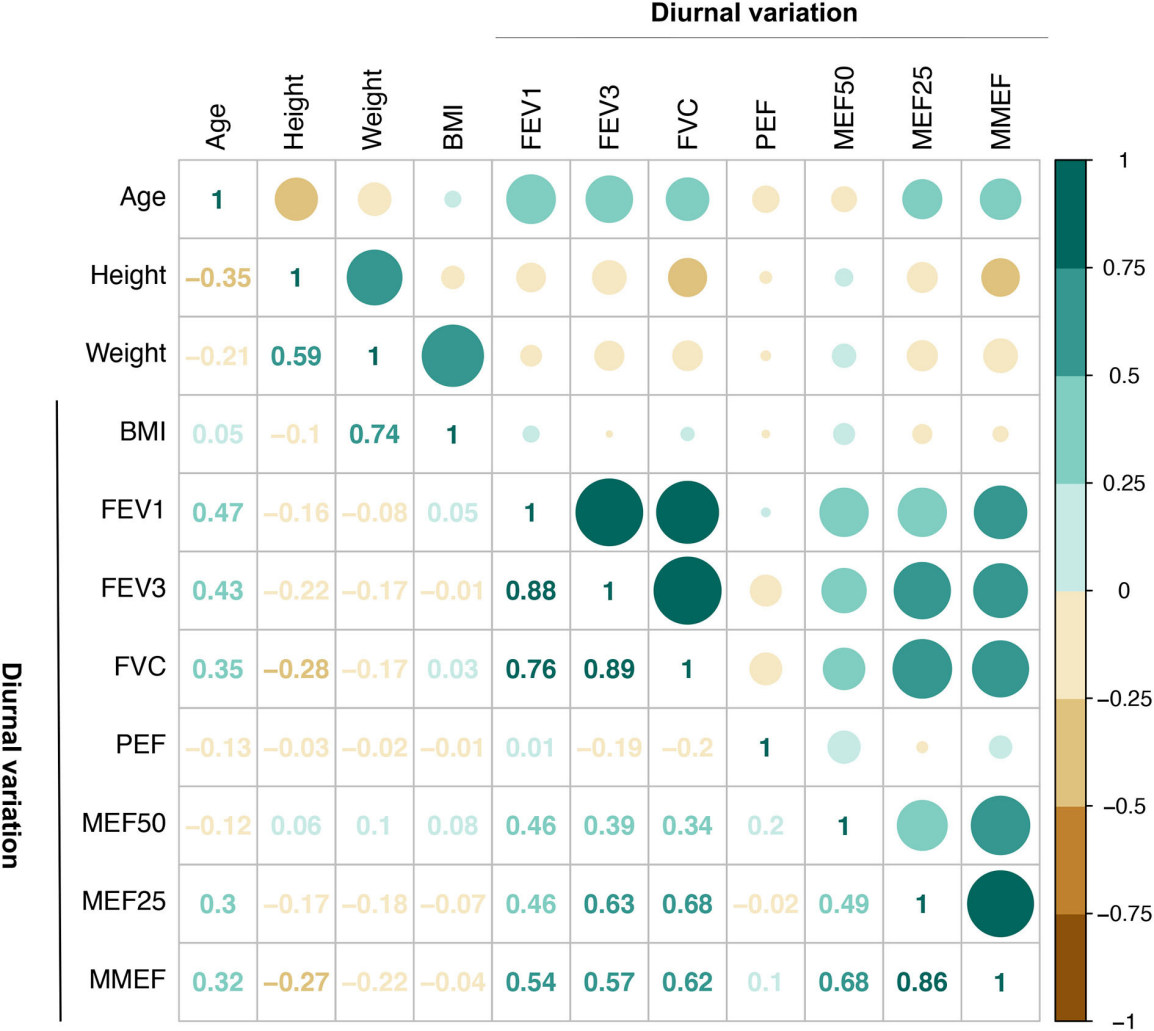
Correlation of diurnal variation of pulmonary function variable values with age, height, weight, and BMI (*N* = 30). A strong relationship between age and FEV_1_ as well as FEV_3_ (*r* = 0.47, *P* = 0.0109 for FEV_1_ and *r* = 0.43, *P* = 0.0215 for FEV_3_) was found. There was no significance between age and FVC, PEF, MEF50, MEF25, and MMEF (*P* > 0.05 for all). No significant correlations were also found among height, weight, or BMI and both large- and small-airway function variables (*P* > 0.05 for all). BMI, body mass index; FVC, forced vital capacity; FEV_1_, forced expiratory volume in 1 s; FEV_3_, FEV in 3 s; MEF50, forced expiratory flow at 50% of forced vital capacity; MEF25, forced expiratory flow at 75% of forced vital capacity; MMEF, forced expiratory flow between 25 and 75%; PEF, peak expiratory flow.

**Figure 6:**
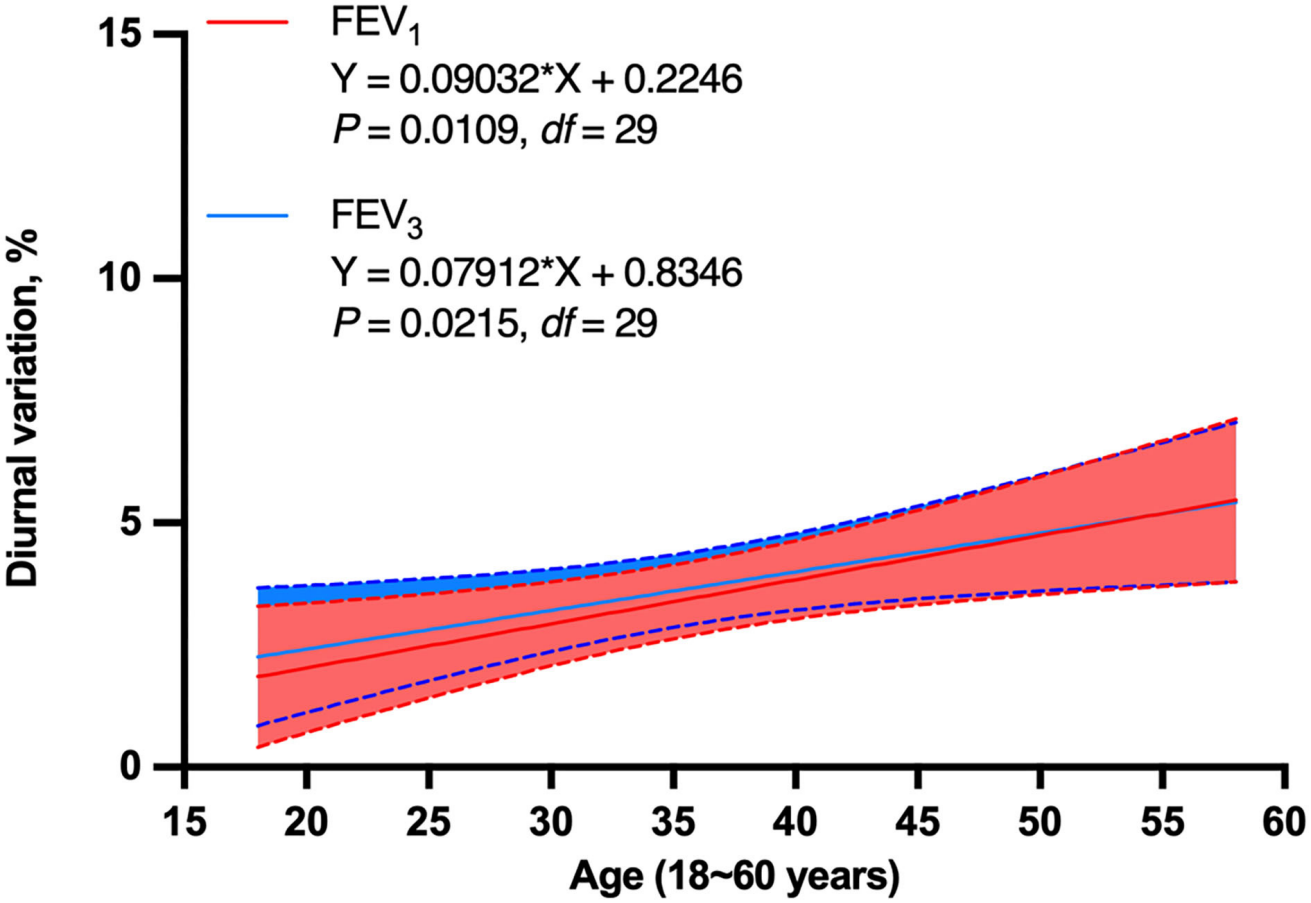
Simple linear regression of diurnal variation of FEV_1_ and FEV_3_ with age in non-smoking healthy adults (*N* = 30). Diurnal variation of FEV_1_ and FEV_3_ were positively related to age in non-smoking healthy adults (*P* = 0.0109, *df* = 29 for FEV_1_ and *P* = 0.0215, *df* = 29 for FEV_3_). FEV_1_, forced expiratory volume in 1 s; FEV_3_, FEV in 3 s; *df* , degree of freedom.

Diurnal variations of FEV_1_ and FEV_3_ were positively related to age (*P* = 0.0109, *r* = 0.47, *df* = 29 for FEV_1_ and *P* = 0.0215, *r* = 0.43, *df* = 29 for FEV_3_, Figures 5, 6). There was no significance between age and FVC, PEF, MEF50, MEF25, and MMEF (*P* > 0.05 for all, Figure 5). No significant correlations were found between height, weight, or BMI and both large- and small-airway function variables (*P* > 0.05 for all, Figure 5).

Diurnal variation of variables in the different age subgroups has also been analyzed. Age-related differences were found, but no statistical significance in large- and small-airway function variables was demonstrated (*P* > 0.05 for all, Table 4).

**Table 4:** Comparison of the diurnal variation of large- and small-airway function variables according to age.

**Variables**	**Age, years**			**All subjects**	***P***
	**18–30**	**30–45**	**45–60**		
*N*	10	10	10^#^	30	–
FVC, %	3.002 (1.019)	4.137 (2.394)	4.700 (2.883)	3.940 (2.275)	0.244
FEV_1_, %	2.434 (1.207)	3.279 (1.411)	4.713 (2.972)	3.482 (2.196)	0.058
FEV_3_, %	2.842 (1.195)	3.222 (1.526)	4.955 (2.741)	3.688 (2.107)	0.053
PEF, %	5.035 (1.997)	5.230 (1.901)	4.031 (2.074)	4.749 (1.996)	0.377
MEF50, %	8.788 (3.004)	7.030 (2.125)	7.940 (3.639)	7.950 (2.995)	0.458
MEF25, %	11.99 (5.250)	16.20 (9.404)	19.33 (15.35)	15.83 (10.93)	0.334
MMEF, %	7.237 (3.112)	8.486 (5.067)	10.47 (5.126)	8.741 (4.561)	0.288

FVC, forced vital capacity; FEV_1_, forced expiratory volume in 1 s; FEV_3_, FEV in 3 s; FEV_3_, FEV in 6 s; MEF50, forced expiratory flow at 50% of forced vital capacity; MEF25, forced expiratory flow at 75% of forced vital capacity; MMEF, forced expiratory flow between 25 and 75%; PEF, peak expiratory flow; SD, standard deviation.

# Four sets of spirometry data from one person did not conform to the acceptability and repeatability criteria and excluded from analysis of diurnal variation.

### Professional spirometry training improved the performance in spite of the age, sex, height, weight, BMI, and education degree

The subjects had lower FEV_1_ (Δ = 0.117 L, *P* < 0.001), FVC (Δ = 0.117 L, *P* < 0.001), FEV_3_ (Δ = 0.121 L, *P* < 0.001), and MEF50 (*P* < 0.01) before training than using the Jaeger spirometer (Supplementary Figure 3). A significant improvement of accuracy in FVC, FEV_1_, FEV_3_, and MEF50 was found, which was consistent with that using a Jaeger spirometer (Figure 5, *P* > 0.05 for all). PEF, MEF25, and MMEF were also consistent with those of a Jaeger spirometer (Supplementary Figure 3, *P* > 0.05 for all).

No significant relationships were found between improvement values, including both large- and small-airway variables, and age, height, weight, or BMI (*P* > 0.05 for all, Supplementary Figure 4). Furthermore, there were no sex- and education degree-related differences between the subgroups for both large- and small-airway variables (*P* > 0.05 for all, Supplementary Figures 5, 6).

## Discussion

To our best knowledge, no relevant studies have been published regarding the influencing factors of both large- and small-airway function variation features using a portable spirometer for 7 days (including morning and evening values) in non-smoking healthy adults. What is new about our study: (1) using a portable spirometer for 7 days (including morning and evening values) to monitor large- and small-airway function in non-smoking healthy adults; (2) investigating factors affecting the morning and evening variability of central and peripheral airway function; and (3) evaluating whether professional training could improve performance of the portable spirometer and possible factors. We found that (1) age, sex, height, and weight all influenced the spirometry values, especially in absolute values; (2) there was a drop until about 35 years of age, followed by a very small but steady increase till 50 years of age, and then a rapid decline in both large- and small-airway function till old age; (3) age was the main influencing factor of both central and peripheral airway function variability, especially for the small-airway function in the evening after 45 years; and (4) professional spirometry training improved the performance in spite of age, sex, height, weight, BMI, and education degree.

Pulmonary function varies with age, standing height, sex, and ethnicity (25). In our study, we also analyzed the influencing factors of home-monitoring spirometry values and validated the appreciable impact of sex, age, height, and weight on both large- and small-airway function variable values, especially on absolute values. Therefore, spirometry results need to be compared with predicted values or FVC, and lower and upper limits of normal (LLN and ULN, respectively) that are appropriate for the individuals being tested. Furthermore, we found no morning and evening differences relate to those influences on lung function. Height was proved more strongly correlated with both large- and small-airway function; however, the effect of height and weight was significantly reduced when BMI was calculated.

There was a steady age-related decline in lung function in adults, which was in concordance with previous findings (24). We discovered that a drop in lung function begins at 25 years of age, followed by a rapidly decline in both large- and small-airway function from about 50 years of age, which is similar to previous research results that show there is an accelerating cross-sectional decline in FEV_1_ after age 30 years in Caucasian adult men, with a nadir at age 62 years when the annual loss ranges between 32 and 46 ml (24), indicating lung function protection may need to start more earlier and aging may contribute to this rapid decline in lung function.

In our previous study, we had reported the healthy individuals' baseline and variation features of large- and small-airway function, low intra-individual variations of central and small-airway variables both in morning and evening, and a slightly higher value of evening variation relative to the corresponding morning variation value. Through the data reanalysis, we found that the CVs of both large- and small-airway function variable values were most affected by age. The CV of lung function values is relatively stable in the 18- to 30-year and 30- to 45-year groups, but significantly increased after 45 years of age, suggesting the LLN is not constant but varies depending on age, especially for the 45- to 60-year group. Then, the correlation of CVs of morning and evening lung function values was calculated separately. Among the CVs of morning lung function values, only the CV of FEV_3_/FVC was strongly positively related to age, indicating that FEV_3_/FVC, which is an indicator of mild airway obstruction and mild lung injury (25–27), may be more sensitive to detect the age-related early airway obstruction than FEV_1_/FVC. Dramatically higher CVs of small-airway function variables (MEF50, MEF25, MMEF, and MMEF/FVC) at night were observed after 45 years of age, suggesting people older than 45 years should pay more attention to monitoring small-airway function in the evening, which will be helpful for early clinical detection of those at high risk for asthma.

Spearman correlation and linear regression analysis exhibited that the diurnal variation of FEV_1_ and FEV_3_ dramatically increased with age, which further verified the influence of age on circadian variation of lung function in the healthy population. Diurnal variation of lung function variables was further explored across age stratification. There was no significant difference in diurnal variation of lung function among age-groups; however, an increasing trend with age was observed. In addition to that, the diurnal variation difference of FEV_1_ and FEV_3_ in different age-groups was very close to significance (*P* = 0.058 for FEV_1_ and = 0.053 for FEV_3_), indicating diurnal variation of these two variables increased with age, especially for the 45- to 60-year population. One possible explanation for these inconsistencies may be related to the small sample size, especially considering the multiple age strata, which included only 30 healthy adult subjects. The sample size limitation in the current study was mainly due to the difficulty in recruitment. It was very difficult to recruit healthy non-smoking adults for continuously monitoring lung function in the morning and at night for 7 days, especially because gender, age, and education degree were homogenized simultaneously. Also, based on the results of this study, we will further carry out a multicenter prospective study to monitor large- and small-airway function with a larger sample size, longer duration, and more time points so as to obtain more accurate reference values of both large- and small-airway function variability in the healthy population of China.

We further explored the improvement of portable spirometer performance after professional training. In our study, lower mean values for FVC, FEV_1_, and FEV_3_ were observed in the GOSPT2000 test, which was in concordance with previous findings (20–22). Nevertheless, those differences were all dispelled after professional training, proving more accuracy and clinical stability of portable spirometer testing than traditional Jaeger spirometer testing. A significant advantage of the GOSPT2000 spirometer over several validated portable spirometers is that both large- and small-airway function variables can be measured (20, 23–27). The values of small-airway function measured by using a portable spirometer were more consistent with those of a Jaeger spirometer in this study, indicating more accuracy of small-airway function variable values of the portable spirometer. Moreover, the improvement was observed after professional spirometry training independent of age, sex, height, weight, BMI, and education degree, indicating the high universality and operability of the portable spirometer, and the results showed that it can be widely used in home settings and primary hospitals.

There are several limitations to our study. First, our study did not include the pediatric population 5–18 years and older population aged more than 60 years. Second, the sample size in our current study is relatively small, and the collection of large numbers of lung function test results in lifelong healthy non-smokers should be performed to ensure the accuracy of the inspection results.

In conclusion, morning and evening lung function values, calculated separately, and sex, age, height, and weight all influenced the spirometry values, especially in absolute values. There was a drop in both large- and small-airway function at about 25 years of age, followed by a rapid decline at about 50 years. Age was the main influencing factor of both central and peripheral airway function variability, especially for the small-airway function in the evening after 45 years. People older than 45 years should pay more attention to monitoring small-airway function in the evening. Education and training improved the portable spirometer accuracy in both large- and small-airway variables independent of age, sex, BMI, and education degree.

## Data availability statement

The raw data supporting the conclusions of this article will be made available by the authors, without undue reservation.

## Ethics statement

The studies involving human participants were reviewed and approved by Ethics Committee of Shanghai General Hospital, Shanghai Jiao Tong University (No. 2021KY073). Written informed consent for participation was not required for this study in accordance with the national legislation and the institutional requirements.

## Author contributions

XZ, YZha, YZho, WB, and MZ conceptualized and designed the study, verified the data, and drafted the manuscript. XZ, YZha, and YZho searched the literature. DY, CL, JL, and YZho acquired the data. XZ independently performed the statistical analyses. WB and MZ verified the statistical analyses. An external mathematical statistician verified all data and statistical analyses. All authors analyzed, visualized, interpreted the data, critically revised, reviewed, and approved the manuscript.

## Funding

The study was supported by the National Natural Science Foundation of China (Grant Nos. 81873402 and 81800020), Appropriate Technique Application Program of Shanghai Municipal Health System (Grant No. 2019SY042), and Scientific and Technological Innovation Program funded by the Science and Technology Commission of Shanghai Municipality (Grant No. 20Y11902400).

## Conflict of interest

The authors declare that the research was conducted in the absence of any commercial or financial relationships that could be construed as a potential conflict of interest.

## Publisher's note

All claims expressed in this article are solely those of the authors and do not necessarily represent those of their affiliated organizations, or those of the publisher, the editors and the reviewers. Any product that may be evaluated in this article, or claim that may be made by its manufacturer, is not guaranteed or endorsed by the publisher.

## Abbreviations

BMI: body mass index
CI: confidence interval
CV: coefficient of variation
df: degrees of freedom
MEF50: forced expiratory flow at 50% of forced vital capacity
MEF25: forced expiratory flow at 75% of forced vital capacity
MMEF: forced expiratory flow between 25 and 75%
FVC: forced vital capacity
FEV_1_: forced expiratory volume in 1 s
FEV_3_: forced expiratory volume in 3 s
HRCT: high-resolution computerized tomography
IQR: interquartile range
LLN: lower limit of normal
PEF: peak expiratory flow
SD: standard deviation
ULN: upper limits of normal.
