# Supplementary material for: Age-related circadian rhythm and variability of large- and small-airway function in healthy non-smoking adults: Data from 7-day diurnal and nocturnal home monitoring using an electronic portable spirometer
Source: Front Public Health. 2022 Oct 17;10:946988. doi: 10.3389/fpubh.2022.946988 (PMC9618715; doi:10.3389/fpubh.2022.946988)
Supplement: Supplementary file 3 [file Data_Sheet_1.docx]

**Age related circadian rhythm and variability of large- and small- airway function in healthy nonsmoking adults**: data from 7-day **diurnal and nocturnal** home monitoring with electronic portable spirometer

Xue Zhang^#^, Yingying Zhang^#^, Yan Zhou^#^, Dongning Yin, Chengjian Lv, Jinwang Lin, Wuping Bao^*^, Min Zhang^*^

Department of Respiratory and Critical Care Medicine, Shanghai General Hospital, Shanghai Jiao Tong University School of Medicine, Shanghai, China

^#^These authors contributed equally to this work and are considered as co-first authors of the publication.

^*^These authors contributed equally to this work and are considered as co-correspondence authors of the publication.

Correspondence:

Min Zhang, PhD, MD, Department of Respiratory and Critical Care Medicine, Shanghai General Hospital, Shanghai Jiao Tong University School of Medicine, 100 Haining Road, Hongkou District Shanghai, China, 200080; E-mail: [maggie_zhangmin@163.com;](mailto:maggie_zhangmin@163.com;) Telephone number: 0086 21 63071428

Wuping Bao, MD, Department of Respiratory and Critical Care Medicine, Shanghai General Hospital, Shanghai Jiao Tong University School of Medicine, 100 Haining Road, Hongkou District Shanghai, China, 200080; E-mail: [wupingbao1982@163.com;](mailto:wupingbao1982@163.com;) Telephone number: 0086 21 63071428

**eTable 1. *P* values for the Spearman correlation** **of pulmonary function (total, morning, and evening) with age, height, weight, and BMI.**

**See Excel format supplemental eTable 1 for details.**

FVC, Forced vital capacity; FEV_1_, Forced expiratory volume in 1 second; FEV_3_, FEV in 3 seconds; MEF50: Forced expiratory flow at 50% of forced vital capacity; MEF25: Forced expiratory flow at 75% of forced vital capacity; MMEF: Forced expiratory flow between 25% and 75%; PEF, Peak expiratory flow; SD, Standard deviation; CI, Confidence interval; t-, total; m-, morning; e-, evening.

**eTable 2. *P* values for the Spearman correlation of pulmonary function (total, morning, and evening) with age, height, weight, and BMI.**

**See Excel format supplemental eTable 2 for details.**

FVC, Forced vital capacity; FEV_1_, Forced expiratory volume in 1 second; FEV_3_, FEV in 3 seconds; MEF50: Forced expiratory flow at 50% of forced vital capacity; MEF25: Forced expiratory flow at 75% of forced vital capacity; MMEF: Forced expiratory flow between 25% and 75%; PEF, Peak expiratory flow; SD, Standard deviation; CI, Confidence interval; t-, total; m-, morning; e-, evening.

**eTable 3. *P* values for the Spearman correlation of diurnal variation of pulmonary function with age, height, weight, and BMI (N= 30).**

| Variables | Age | Height | Weight | BMI | FEV_1_ | FEV_3_ | FVC | PEF | MEF50 | MEF25 | MMEF |
| --- | --- | --- | --- | --- | --- | --- | --- | --- | --- | --- | --- |
| Age | 1 |  |  |  |  |  |  |  |  |  |  |
| Height | 0.0634 | 1 |  |  |  |  |  |  |  |  |  |
| Weight | 0.2817 | 0.0007 | 1 |  |  |  |  |  |  |  |  |
| BMI | 0.8052 | 0.6182 | *** | 1 |  |  |  |  |  |  |  |
| FEV_1_ | 0.0109 | 0.4088 | 0.6707 | 0.8022 | 1 |  |  |  |  |  |  |
| FEV_3_ | 0.0215 | 0.2522 | 0.3921 | 0.9728 | *** | 1 |  |  |  |  |  |
| FVC | 0.0595 | 0.1379 | 0.3769 | 0.8685 | *** | *** | 1 |  |  |  |  |
| PEF | 0.4877 | 0.8960 | 0.9314 | 0.9581 | 0.9453 | 0.3328 | 0.3060 | 1 |  |  |  |
| MEF50 | 0.5383 | 0.7673 | 0.5888 | 0.6640 | 0.0122 | 0.0386 | 0.0739 | 0.2923 | 1 |  |  |
| MEF25 | 0.1162 | 0.3664 | 0.3583 | 0.7221 | 0.0123 | 0.0003 | *** | 0.9091 | 0.0068 | 1 |  |
| MMEF | 0.0911 | 0.1493 | 0.2495 | 0.8259 | 0.0024 | 0.0013 | 0.0003 | 0.6216 | *** | *** | 1 |

***: < 0.001

BMI, Body mass index; FVC, Forced vital capacity; FEV_1_, Forced expiratory volume in 1 second; FEV_3_, FEV in 3 seconds; MEF50: Forced expiratory flow at 50% of forced vital capacity; MEF25: Forced expiratory flow at 75% of forced vital capacity; MMEF: Forced expiratory flow between 25% and 75%; PEF, Peak expiratory flow.

**eFigure 1. Difference of large- and small- airway function variables values according to sex.**


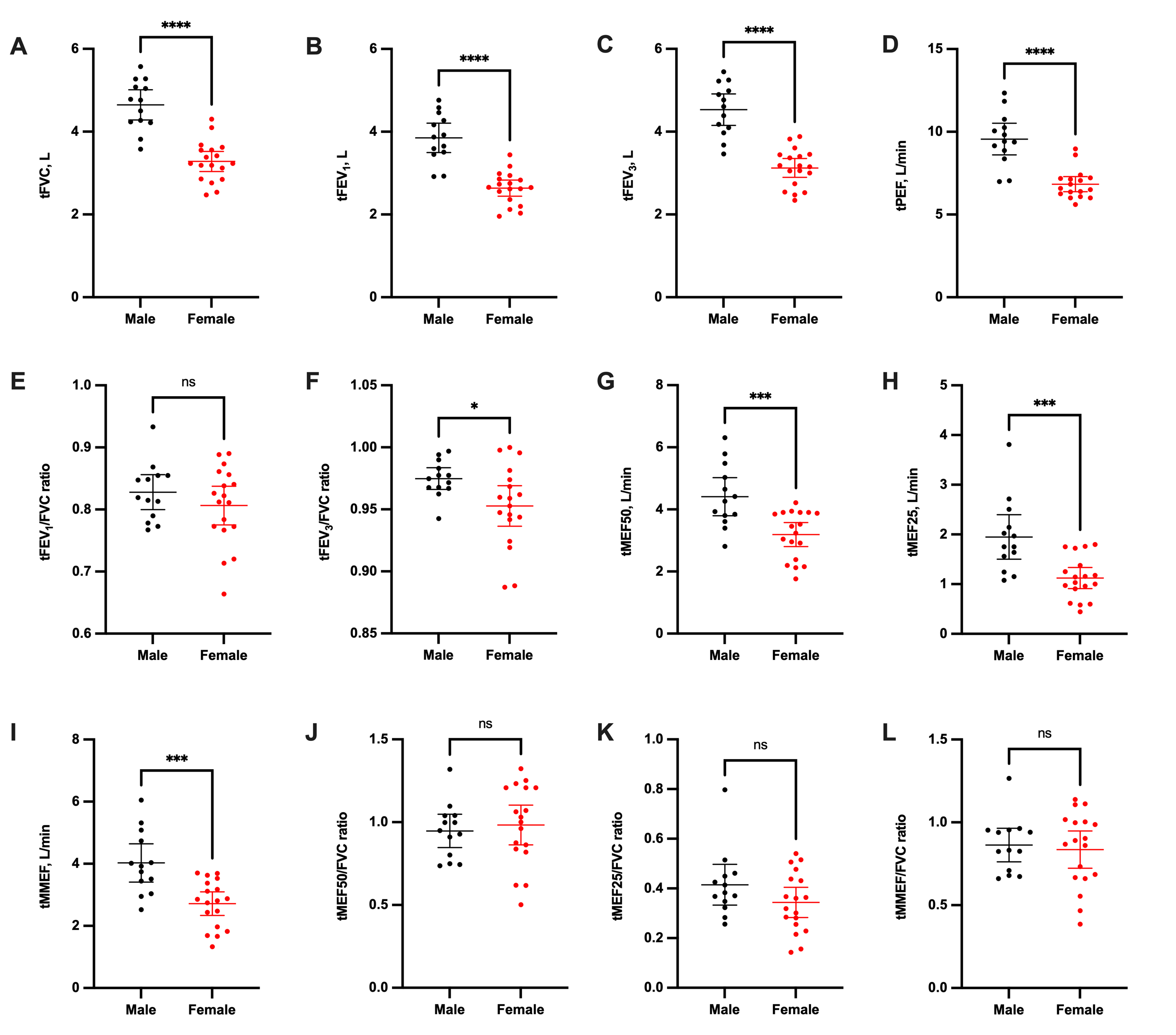


Both large- and small- airway function absolute values of male adults were dramatically higher than that of female adults (eFigure 1), however, FEV_1_/FVC (eFigure 1-E) and MEFs/FVC (eFigure 1-J, K, and L) showed no difference between male and female subgroups (*P* > 0.05).

FVC, Forced vital capacity; FEV_1_, Forced expiratory volume in 1 second; FEV_3_, FEV in 3 seconds; MEF50: Forced expiratory flow at 50% of forced vital capacity; MEF25: Forced expiratory flow at 75% of forced vital capacity; MMEF: Forced expiratory flow between 25% and 75%; PEF, Peak expiratory flow.

**eFigure 2. Difference of CV of large- and small- airway function variables according to sex.**

**
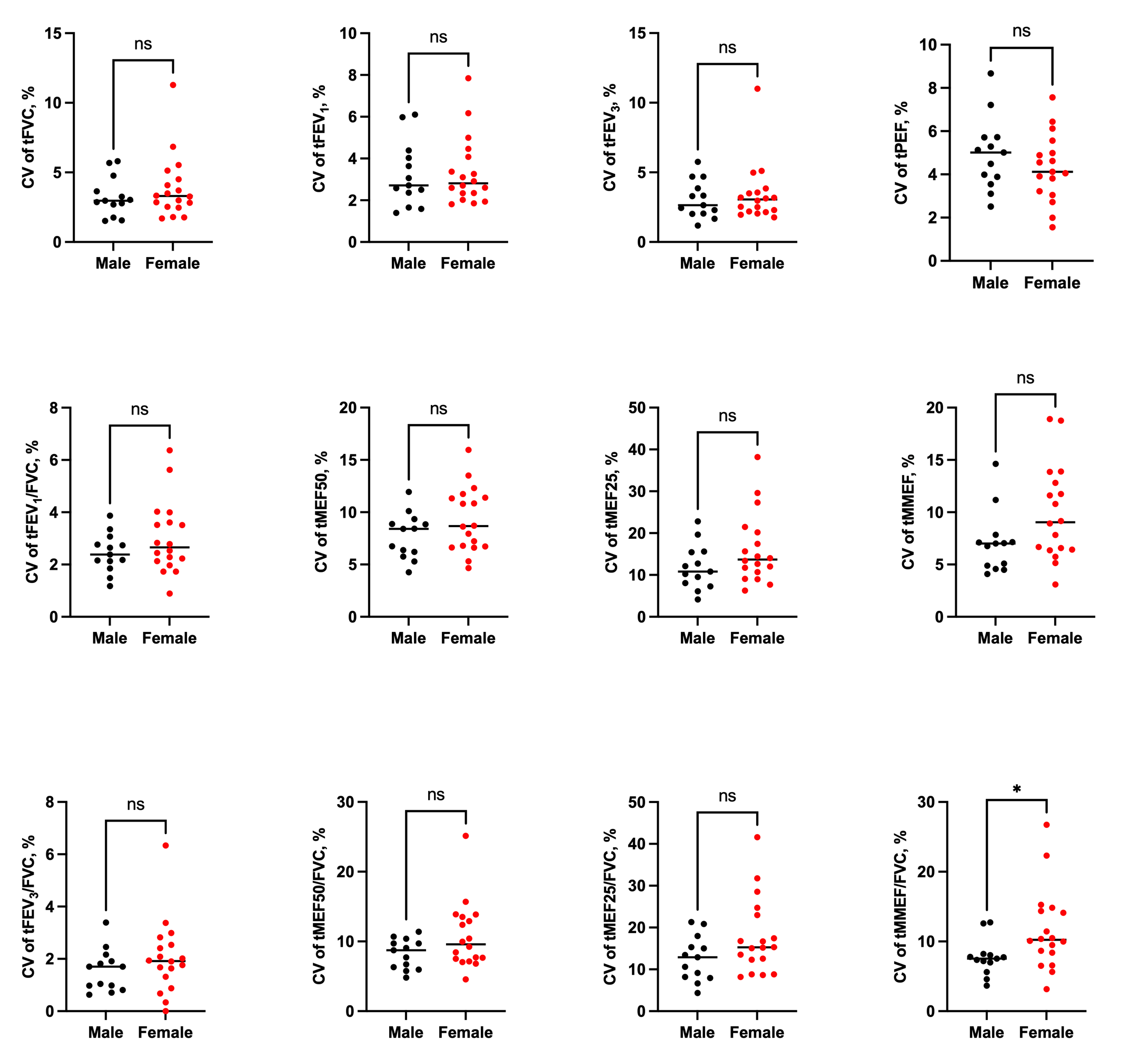
**

Except for CV of MMEF/FVC (*P* < 0.05), there was also no significant difference across sex (*P* > 0.05 for all).

CV, Coefficient of variation; FVC, Forced vital capacity; FEV_1_, Forced expiratory volume in 1 second; FEV_3_, FEV in 3 seconds; MEF50: Forced expiratory flow at 50% of forced vital capacity; MEF25: Forced expiratory flow at 75% of forced vital capacity; MMEF: Forced expiratory flow between 25% and 75%; PEF, Peak expiratory flow.

**eFigure 3. Influence of training to assessment of variables by GOSPT2000.**

**
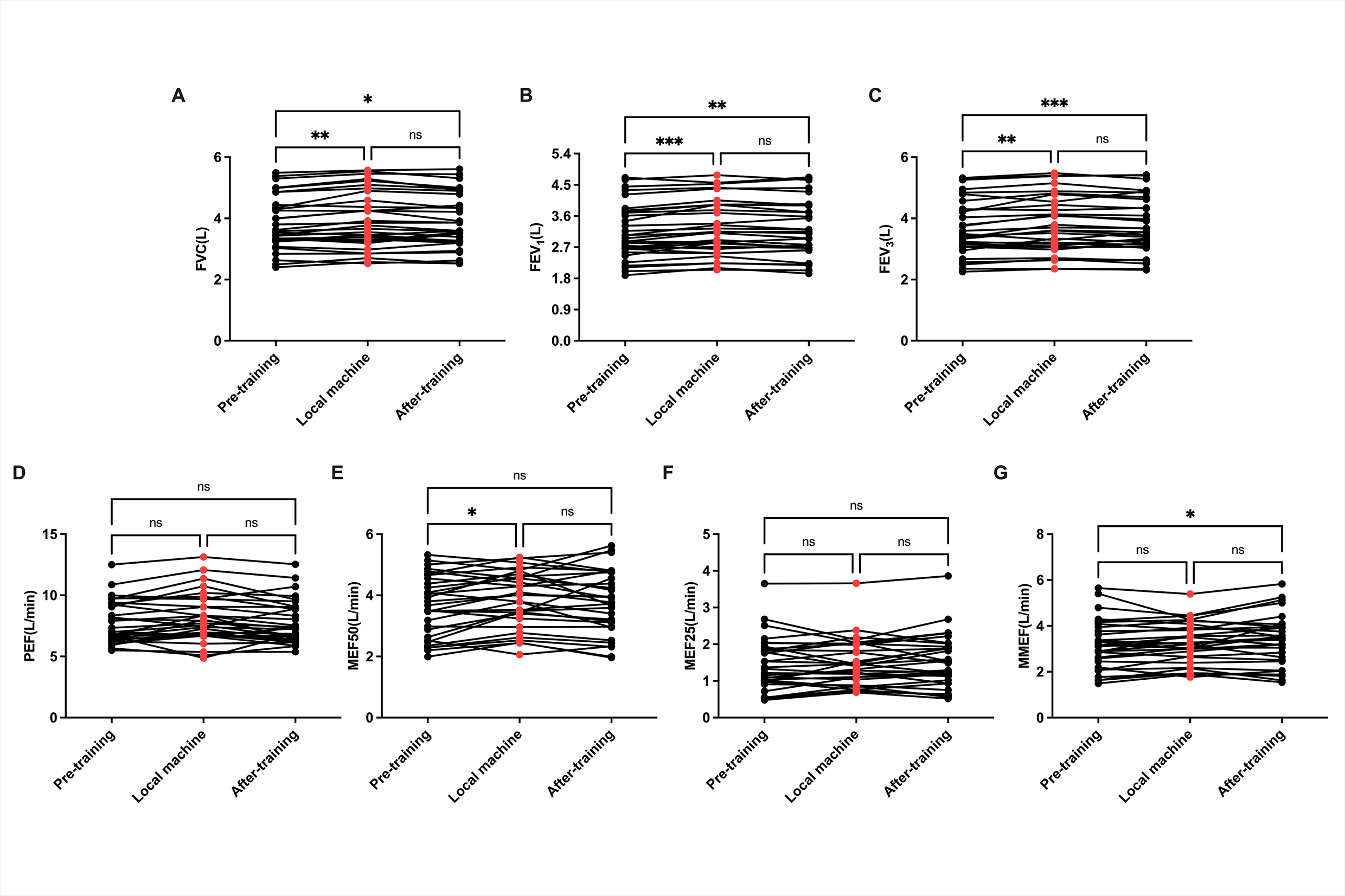
**

Subjects got lower FEV_1_ (∆=0.117 L, *P* <.001), FVC (∆=0.117 L, *P* <.001), FEV_3_ (∆= 0.121 L, *P* <.001), and MEF50 (*P* <.05) before training, compared with Jaeger spirometer. There were no significant inter-group differences in PEF, MEF25 and MMEF (*P >*.05 for all). A significant improvement of accuracy in FVC, FEV_1_, FEV_3_, and MEF50 (*P >*.05 for all) were confirmed after training. PEF, MEF25 and MMEF remains consistent with that of Jaeger spirometer.

FVC, Forced vital capacity; FEV_1_, Forced expiratory volume in 1 second; FEV_3_, FEV in 3 seconds; MEF50: Forced expiratory flow at 50% of forced vital capacity; MEF25: Forced expiratory flow at 75% of forced vital capacity; MMEF: Forced expiratory flow between 25% and 75%; PEF, Peak expiratory flow.

**eFigure 4. Professional spirometry training improved the** **performance in spite of the age, sex, height, weight, BMI, and education degree.**

**
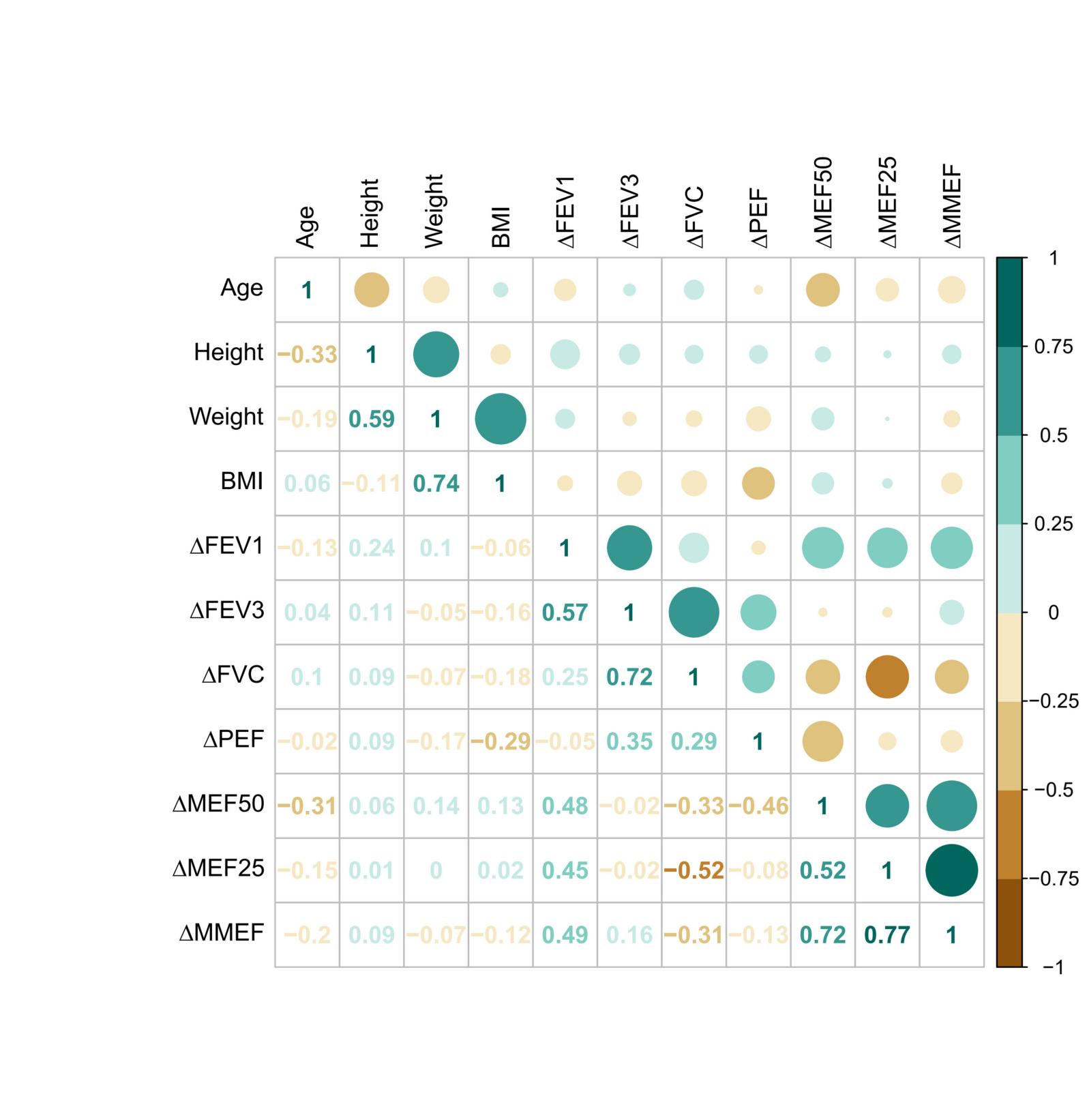
**

No significant relationships were found between improvement values, including both large- and small- airway variables, and age, height, weight, or BMI (*P* > 0.05).

BMI, Boy mass index; FVC, Forced vital capacity; FEV_1_, Forced expiratory volume in 1 second; FEV_3_, FEV in 3 seconds; MEF50: Forced expiratory flow at 50% of forced vital capacity; MEF25: Forced expiratory flow at 75% of forced vital capacity; MMEF: Forced expiratory flow between 25% and 75%; PEF, Peak expiratory flow.

**eFigure 5.** **Professional spirometry traini****ng improved the** **performance in spite of the** **sex.**

**
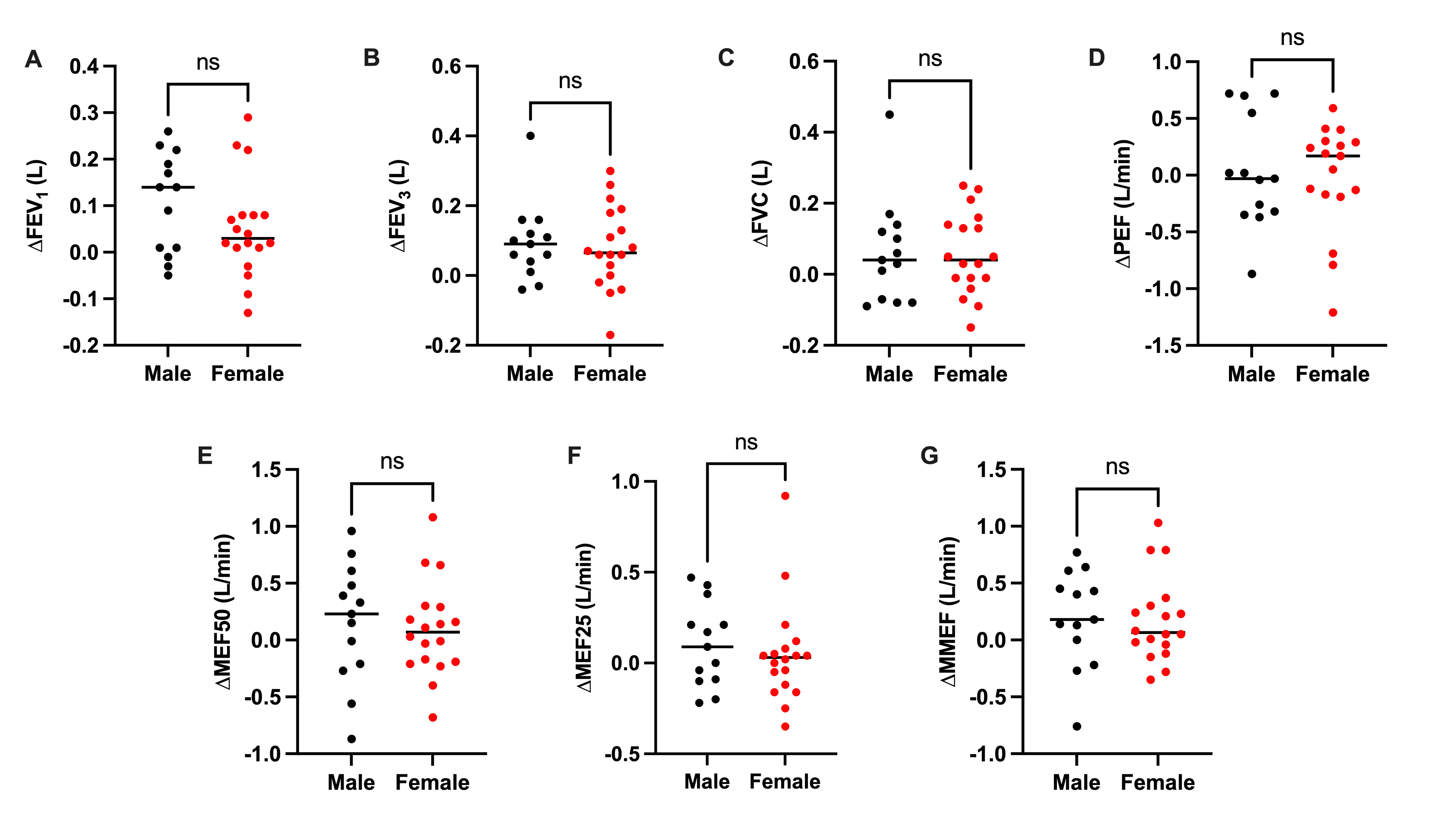
**

FVC, Forced vital capacity; FEV_1_, Forced expiratory volume in 1 second; FEV_3_, FEV in 3 seconds; FEV_3_, FEV in 6 seconds; MEF50: Forced expiratory flow at 50% of forced vital capacity; MEF25: Forced expiratory flow at 75% of forced vital capacity; MMEF: Forced expiratory flow between 25% and 75%; PEF, Peak expiratory flow; SD, Standard deviation.

**eFigure 6. Professional spirometry training improved the** **performance in spite of the education degree.**

**
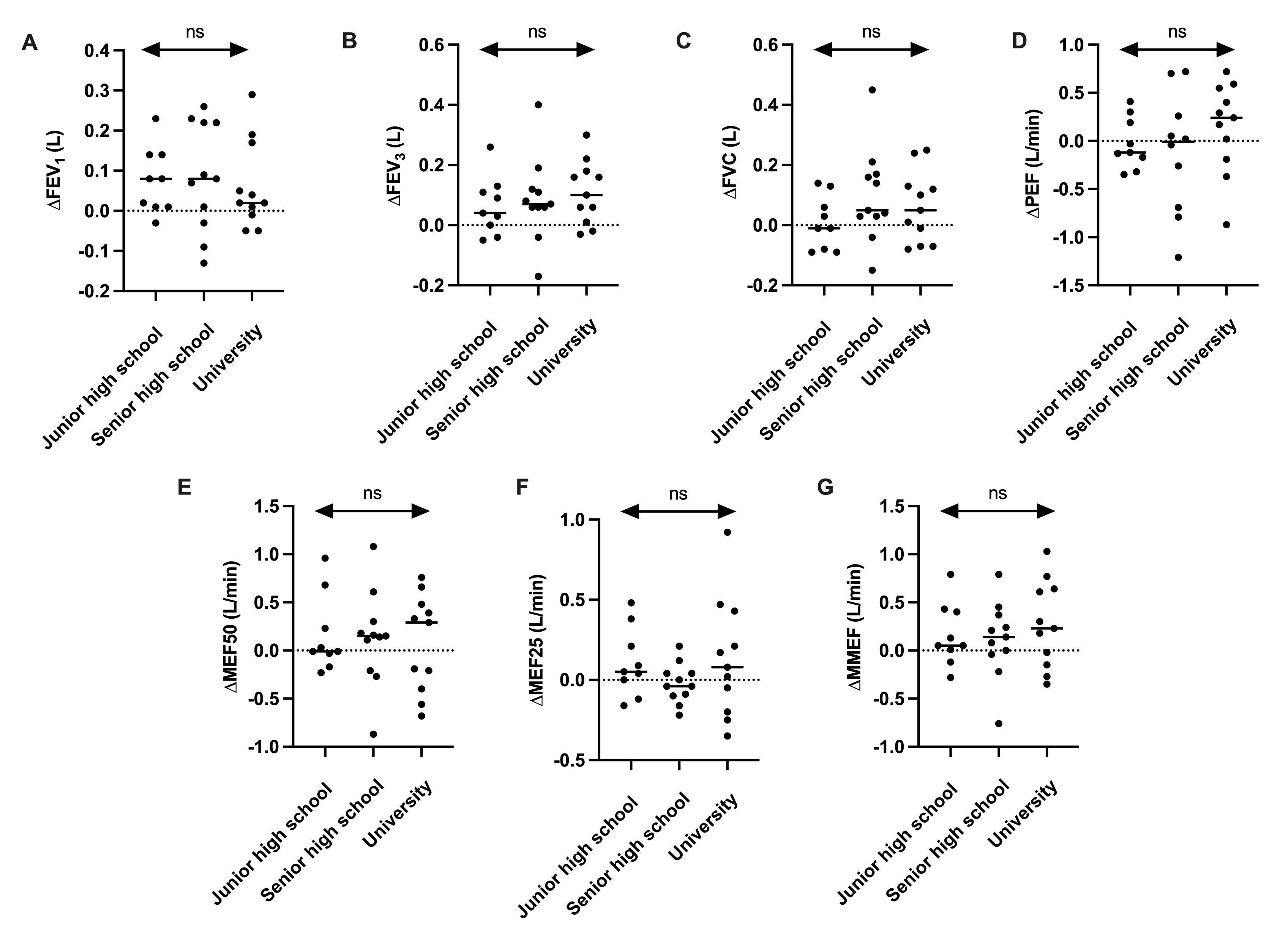
**

FVC, Forced vital capacity; FEV_1_, Forced expiratory volume in 1 second; FEV_3_, FEV in 3 seconds; FEV_3_, FEV in 6 seconds; MEF50: Forced expiratory flow at 50% of forced vital capacity; MEF25: Forced expiratory flow at 75% of forced vital capacity; MMEF: Forced expiratory flow between 25% and 75%; PEF, Peak expiratory flow; SD, Standard deviation.
